# Integrated lipidomics and network pharmacology analysis to reveal the mechanisms of berberine in the treatment of hyperlipidemia

**DOI:** 10.1186/s12967-022-03623-0

**Published:** 2022-09-08

**Authors:** Yuting Chen, Kaipeng Li, Han Zhao, Zhangsen Hao, Yuxin Yang, Mingming Gao, Ding Zhao

**Affiliations:** 1 The Postdoctoral Research Station of Biology, Hebei Medical University, Shijiazhuang, 050017 China; 2 The Department of Pharmacognosy, School of Pharmacy, Hebei Medical University, Shijiazhuang, 050017 China; 3 The Laboratory of Lipid Metabolism, Institute of Basic Medicine, Hebei Medical University, Shijiazhuang, 050017 Hebei China

**Keywords:** Berberine, Hyperlipidemia, Lipidomics, Network pharmacology

## Abstract

**Background:**

Berberine (BBR), an isoquinoline alkaloid isolated from Rhizoma Coptis, is widely used in the treatment of hyperlipidemia (HLP) in China. At present, the efficacy of BBR against HLP is relatively clear, but there are few researches on its mechanism. The purpose of this study was to evaluate the potentially beneficial role of BBR in HLP hamster models, as well as investigate its possible mechanisms and potential lipid biomarkers in combination with network pharmacology.

**Methods:**

HLP hamster model was induced by high-fat diet. Hematoxylin—eosin (HE) staining was used to determine the degree of hepatic pathological injury. Liquid chromatography-mass spectrometry was used to analyze lipid metabolism profiles of liver samples, and multiple statistical analysis methods were used to screen and identify lipid biomarkers. The possible molecular mechanism was unraveled by network pharmacology.

**Results:**

The results showed that 13 metabolites, including CE (16:1), HexCer (D18:1/19:0) and LPC (O-22:0) were biomarkers of BBR regulation. CHPT1, PLA2G4A, LCAT and UGCG were predicted as the lipid-linked targets of BBR against HLP, whilst glycerophospholipid and sphingolipid metabolism were the key pathways of BBR against HLP.

**Conclusions:**

In summary, this study provides new insights into the protective mechanism of BBR against HLP through network pharmacology and lipidomic approaches.

**Supplementary Information:**

The online version contains supplementary material available at 10.1186/s12967-022-03623-0.

## Introduction

Hyperlipidemia (HLP) is a metabolic disease caused by abnormal fat metabolism, which is mainly manifested by an abnormal increase of Total cholesterol (TC), Triglyceride (TG) and low-density lipoprotein-cholesterol (LDL-C) levels in the blood [1]. The incidence of HLP has increased rapidly due to improved lifestyles and an increase in high-energy diets, which has been identified as a major risk factor for various cardiovascular diseases [2]. Therefore, it is particularly important to strengthen the effective prevention and regular treatment of the disease. At present, statins are the first choice for regulating abnormal blood lipids and lowering lipids, but long-term use of statins has muscle toxicity and liver toxicity. Phytotherapy has be considered an interesting tool in the treatment of diseases, which involves the use of active ingredients or plant extracts [3]. The active ingredients of traditional Chinese medicine have attracted increasing attention of researchers due to their advantages of stable curative effect and few side effects.

Berberine (BBR) is an isoquinoline alkaloid isolated from Coptidis Rhizoma, which has traditionally been used to treat diarrhea. Modern pharmacological studies have shown that BBR can relieve dyslipidemia and glucose metabolism. BBR has an anti-HLP effect, which enhances LDL receptor (LDLR) expression through stabilizing the LDLR mRNA [4] and suppressing transcription of proprotein convertase subtilisin/kexin type 9 (PCSK9) [5]. BBR could improve insulin resistance and maternal–fetal outcomes of gestational diabetes mellitus rats through modulation of IKK/NF-kB, JNK, and IRS-1/AKT signaling pathways in the liver [6]. BBR could also increase adipose triglyceride lipase in 3T3-L1 adipocytes through the adenosine monophosphate (AMP)-activated protein kinase (AMPK) pathway to maintain lipid homeostasis [7]. Chang demonstrated that BBR could alter circulating ceramides to improve nonalcoholic fatty liver disease [8]. However, there is no characterization of lipid metabolism before and after berberine treatment with HLP.

Lipids are involved in various physiological processes and play an important role in energy conversion, cell signal transduction, cell differentiation and apoptosis [9]. The liver plays a major role in lipid metabolism, and abnormal hepatic lipid metabolism is closely related to the occurrence and development of HLP [10]. Lipidomics is a new omics research technique that can determine lipid composition and identify lipid biomarkers at the molecular level to study the pathogenesis of lipid metabolism-related diseases [11]. Network pharmacology is a new discipline to understand the interaction between drugs and diseases from a new perspective and guide the development of new drugs. Under the guidance of Systems Biology, the network construction method of "drug ingredients-action target-signal pathway-treatment mechanism" is used to intuitively display the pathways and pathways of the effective drug ingredients in treating related diseases, which is helpful to promote the study of the material basis and molecular mechanism of the effectiveness of Traditional Chinese Medicine [12].

In this study, we developed a novel integrated strategy to explore the biological mechanisms of BBR therapy for HLP based on lipidomics and network pharmacology. First, lipid biomarkers and pathways were analyzed by lipidomics and multivariate data analysis based on UHPLC-QTRAP-MS/MS. Secondly, the potential targets of BBR for HLP treatment were predicted through network pharmacology. Finally, the common goals of lipidomics and network pharmacology were analyzed to investigate the therapeutic mechanisms of BBR. This study is helpful to establish the relationship between biomarkers and hub genes, and provides a scientific basis for accurate screening of biomarkers. This study also provides a new idea for the clinical treatment of HLP and the systematic study of the effective ingredients of Traditional Chinese medicine.

## Materials and methods

### Chemicals and reagents

BBR (061002, purity > 98%) was obtained from Sichuan Xieli Pharmaceutical Co. Ltd. (Sichuan, China). Serum biochemistry kits for determining the levels of serum total cholesterol (TC), total triglyceride (TG) and low-density lipoprotein cholesterol (LDL-C) were supplied by Zhongsheng Beikong Biotechnology Co., Ltd. (Beijing, China). Methanol, acetonitrile, isopropanol and formic acid were provided by Merck. (Frankfurt, Germany). The reference standards were purchased from Shanghai ZZ biotechnology Co., Ltd. (Shanghai, China).

### Animal experiments

Golden Syrian Hamsters (120 ± 10 g) were purchased from Hebei INVIVO Biotech Co. Ltd. The animal experiments were processed according to the Guidelines for Animal Experimentation of Hebei INVIVO Biotechnology Co., Ltd. Before the experiment, the animals were put in a temperature friendly room with free food and water for at least a week.

Eighteen male Golden Syrian Hamsters aged 8–10 weeks were randomly selected. After one week of adaptive feeding, they were randomly divided into three groups: Normal-fat diet group (NFD), high-fat diet group (HFD), and BBR administration group (BBR), with six hamsters in each group. The NFD group was fed with standard laboratory rat chow, and the HFD group was fed with diet of 40% fructose, 20% lard, 0.5% cholesterol and 39.5% standard laboratory rat chow (Hebei INVIVO Biotech Co. Ltd). After two weeks of continuous feeding, there were significant differences in serum lipid indexes between NFD group and HFD group, and the HLP model was successfully established. Then the BBR group was given BBR (200 mg/kg) [13, 14] by gavage while feeding on diet of HFD group, once a day for 14 consecutive days. The hamsters in the other groups were given the same dose distilled water by gavage.

### Biochemical indexes and hepatic histopathological analysis

The serum concentrations of total cholesterol (TC), triglyceride (TG) and low-density lipoprotein (LDL-C) were analyzed by automatic biochemical analyzer (CMax Plus). Hematoxylin and eosin staining (H&E) staining was carried out to assess hepatic vacuolization according to a standard procedure of the previous reports [15]. Meanwhile, the frozen sections were stained with Oil Red O (ORO) to further validate quantify lipid droplets.

### Liver lipidomics analysis

Liver tissues (50 mg) were homogenized in a 2 mL lipid extract mixture (methyl tert-butyl ether: methanol = 3:1, V/V), including 54 kinds of internal standard substances (Table 1). After homogenization, the mixture was vortexed for 2 min and then centrifuged at 12,000 rpm at 4 ℃ for 10 min. 200 μL supernatant was absorbed and evaporated to dryness under a stream of N_2_. Finally, the lipid extracts were redissolved in 200 μL lipid complex solution (acetonitrile: isopropanol = 1:9, V/V) for LC–MS/MS analysis. Additionally, the quality control (QC) samples were prepared by mixing equal amounts of supernatant from all samples.

**Table 1:** The internal standard information

Serial number	Classification	Glass	Name	Internal standard concentration μM (μmol/L)
1	Phosphatidylcholine	PC	PC(16:0/16:0)-d9	0.2
2	Oxidized lipids	Eicosanoid	5S-HETE-d8	0.04
3	Free fatty acid	FFA	Arachidonic Acid-d8	0.2
4	Acyl carnitine	CAR	CAR(16:0)-d3	0.04
5	Cholesterol ester	CE	CE(18:1)-d7	2
6	ceramide	Cer	Cer(d18:1/15:0)-d7	0.1
7	ceramide	Cer	Cer(d18:1-d7/16:0)	0.1
8	ceramide	Cer	Cer(d18:1-d7/18:0)	0.1
9	ceramide	Cer	Cer(d18:1-d7/24:0)	0.1
10	ceramide	Cer	Cer(d18:1-d7/24:1)	0.1
11	phytoceramide	Cert	Cer(t18:0/22:0-d3)	0.1
12	Ceramide 1-phosphate	CerP	CerP(d18:1/8:0)	0.4
13	cholesterol	Cho	cholesterol-d7	5
14	Coenzyme Q	CoQ	CoQ10-d9	0.2
15	diglyceride	DG	DG(17:0/17:0)-d5	0.2
16	Free fatty acid	FFA	FFA(16:0)-d31	0.2
17	Bile acid	BA	GCDCA-d4	0.2
18	Sugar sheath fat	HexCer	HexCer(d18:1-d5/18:0)	0.4
19	Lysophosphatidic acid	LPA	LPA(17:0)	0.5
20	Lysophosphatidylcholine	LPC	LPC(15:0)-d5	0.2
21	Lysophosphatidylcholine	LPC	LPC(16:0)-d31	0.2
22	Lysophosphatidylcholine	LPC	LPC(17:0)-d5	0.2
23	Lysophosphatidylcholine	LPC	LPC(18:1)-d7	0.2
24	Lysophosphatidylethanolamine	LPE	LPE(15:0)-d5	0.2
25	Lysophosphatidylethanolamine	LPE	LPE(18:1)-d7	0.2
26	Lysophosphatidyl glycerol	LPG	LPG(15:0)-d5	0.2
27	Lysophosphatidyl glycerol	LPG	LPG(17:1)	0.2
28	Lysophosphatidylinositol	LPI	LPI(17:1)	0.2
29	Lysophosphatidylserine	LPS	LPS(17:1)	0.2
30	monoglyceride	MG	MG(18:1-d7)	0.5
31	Phosphatidic acid	PA	PA(17:0/17:0)	0.5
32	Phosphatidylcholine	PC	PC(15:0/18:1(d7))	0.2
33	Phosphatidylcholine	PC	PC(16:0-d31/18:1)	0.2
34	Phosphatidylcholine	PC	PC(14:0/14:0)-d9	0.2
35	Phosphatidylethanolamine	PE	PE(15:0/18:1(d7))	0.2
36	Phosphatidylethanolamine	PE	PE(16:0-d31/18:1)	0.2
37	Phosphatidylethanolamine	PE	PE(17:0/14:1)-d5	0.2
38	Phosphatidylethanolamine	PE	PE(17:0/18:1)-d5	0.2
39	Phosphatidylethanolamine	PE	PE(17:0–22:4)-d5	0.2
40	Phosphatidyl glycerol	PG	PG(15:0/18:1(d7))	0.4
41	Phosphatidyl glycerol	PG	PG(16:0/d31/18:1)	0.4
42	Phosphatidylinositol	PI	PI(16:0-d31/18:1)	0.2
43	Phosphatidyl methanol	PMeOH	PMeOH(16:0/16:0)	0.1
44	Phosphatidylserine	PS	PS(15:0/18:1(d7))	0.4
45	Phosphatidylserine	PS	PS(16:0(d31)/18:1)	0.4
46	Phosphatidylserine	PS	PS(16:0/16:0)-d9	0.4
47	sphingomyelin	SM	SM(d18:1/18:1)-d9	0.2
48	sphingomyelin	SM	SM(d18:1/20:1)-d9	0.2
49	sphingomyelin	SM	SM(d18:1/22:1)-d9	0.2
50	sphingomyelin	SM	SM(d18:1-d9/15:0)	0.2
51	sphingosine	SPH	SPH(18:1)-d7	0.04
52	triglycerides	TG	TG(14:0/16:1/14:0)-d5	0.2
53	triglycerides	TG	TG(16:0–15:1–16:0)-d5	0.2
54	triglycerides	TG	TG(17:0/17:1/17:0)-d5	0.2

### Chromatography-mass spectrometry acquisition conditions

Data acquisition instrument system mainly includes Ultra Performance Liquid Chromatography (UPLC) (ExionLC™ AD, https://sciex.com.cn/) and Tandem Mass Spectrometry (MS/MS) (QTRAP® 6500^+^, https://sciex.com.cn/).

The liquid chromatography was performed on the Thermo Accucore™C30 column (2.6 μm, 2.1 mm × 100 mm I.D.). Mobile phase A: acetonitrile/water (60/40, V/V) (containing 0.1% formic acid, 10 mmol/L ammonium formate). Mobile phase B: acetonitrile/isopropyl alcohol (10/90, V/V) (containing 0.1% formic acid, 10 mmol/L ammonium formate). Gradient elution procedure: 0–2 min, 20–30%B; 2–4 min, 30–60%B; 4–9 min,60–85%B; 9–14 min, 85–90%B; 14–15.5 min, 90–95%B; 15.5–17.3 min, 95%B; 17.3–17.5 min, 95–20%B; 17.5–20 min, 20%B. The flow rate was 0.35 mL/min. Column temperature was 45 °C. The injection volume was 2 μL.

The mass spectrometry conditions are as follows: Electrospray Ionization (ESI) temperature was 500 ℃, mass spectrometry voltage was 5500 V in positive ion mode and − 4500 V in negative ion mode. Ion sources gas 1, 45 psi; Gas 2, 55 psi; Curtain gas, 35 psi. In the triple quadrupole mass spectrometry, each ion pair was scanned according to the optimized Declustering Potential and Collision Energy. Multiple response monitoring (MRM) was used for qualitative and quantitative analysis of lipid spectrum.

### Target acquisition

In order to discover the targets of BBR, we applied the TCMSP database (https://old.tcmsp-e.com/tcmsp.php), Swiss Target Prediction (http://www.swisstargetprediction.ch/), Pubchem Server (https://pubchem.ncbi.nlm.nih.gov/) and PharmMapper Server (http://www.lilab-ecust.cn/pharmmapper/). In addition, the related disease targets were searched and analyzed by inputting the keywords “Hyperlipidemia” into GeneCards Database (http://www.genecards.org/), DrugBank (https://go.drugbank.com/), OMIM Database (http://omim.org/) and DisGeNET database (https://www.disgenet.org/). Then, the false-positive and duplicate targets were deleted and integrated. Finally, the targets of BBR and the targets of HLP were intersected to obtain the potential targets of BBR treating HLP.

### Network construction

The potential lipid biomarkers regulated by BBR were imported into Metscape to generate a metabolite-reaction-enzyme-gene network. The predicted targets of BBR and lipid targets were combined to import into STRING 11.5 database, the minimum interaction threshold was set as “Highest confidence > 0.9” to obtain the protein–protein interaction (PPI) network. Based on the PPI results, we focused on their linked targets to discover the underlying mechanisms. Thus, the network containing relationships among BBR, metabolites and related genes was established in Metscape. Through the analysis of this network, key targets in the interaction network were screened for analysis [16].

### Further validation of the targets

Immunohistochemical methods were used to further validate the targets in the liver of each group of hamsters. After the liver tissue was deparaffinized, it was immersed in xylene I and xylene II for 5 min in sequence, hydrated in gradient alcohol for 10 min each, and antigen was recovered at a high temperature. Then, 3% H_2_O_2_ was added dropwise to incubate the specimen in a humidified chamber at room temperature for 30 min. The goat serum working solution was added for blocking dropwise and incubated in a humidified box at room temperature for 40 min. Add the primary antibodies CHPT1 (1:100, bioss), LCAT (1:200, servicebio), PLA2G4A (1:200, servicebio) and UGCG (1:100, ABclonal) respectively, and place them in a humid chamber at 4 ºC and incubate overnight. Add biotin working solution dropwise, and incubate in a humid chamber at room temperature for 35 min. Streptomycin working solution labeled with horseradish peroxidase was added and incubated for 35 min in a wet box at ambient temperature. After DAB color rendering, dehydration and transparency, the tissue sections were fixed with neutral gum and analyzed by light microscopy. The mean optical density was quantified by Image-Pro Plus 6.0 software.

### Statistical methods

**Fig. 1:**
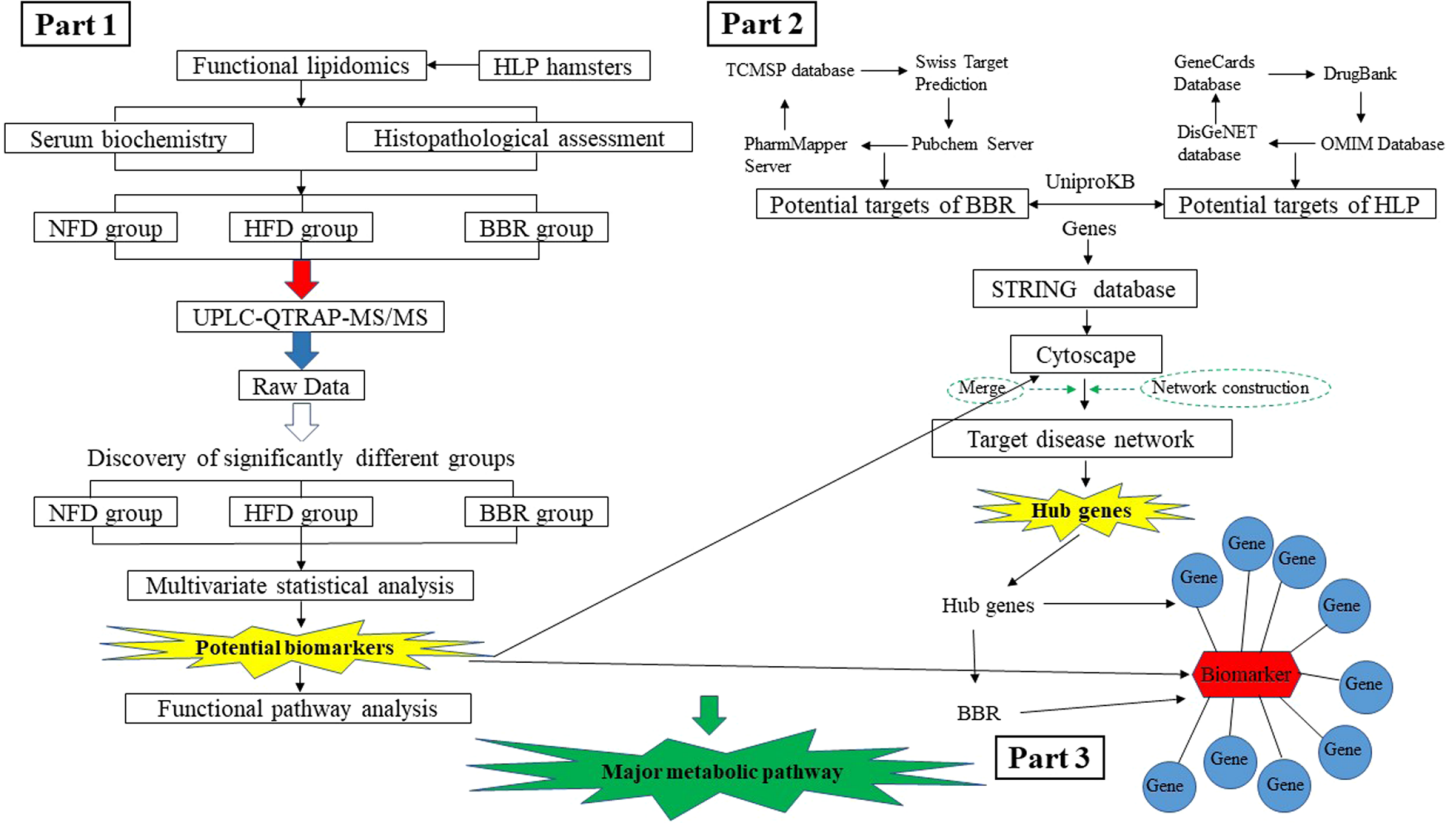
Flowchart of the integrated strategy platform. Part 1: The mechanism of berberine (BBR) in the treatment of hyperlipidemia (HLP) was comprehensively described based on targeted lipidomics. Part 2: The network pharmacology approach was developed to identify targets of BBR for the treatment of HLP. Part 3: Potential links between biomarkers and hub genes

All data were expressed as mean ± standard deviation (X ± SD). GraphPad Prism 8.0 software was used for graphing and SPSS26.0 software was used for statistical analysis. Differences between groups were compared by one-way ANOVA, and the criterion of p < 0.05 was adopted for statistical significance. The screening criteria for significantly different lipid metabolites were fold change (FC ≥ 2, FC ≤ 0.5, VIP > 1 and *p* < 0.05). A flow chart of the scheme for this research is shown in Fig. 1.

## Results

### Analysis of Biochemical indexes and body weight

**Fig. 2:**
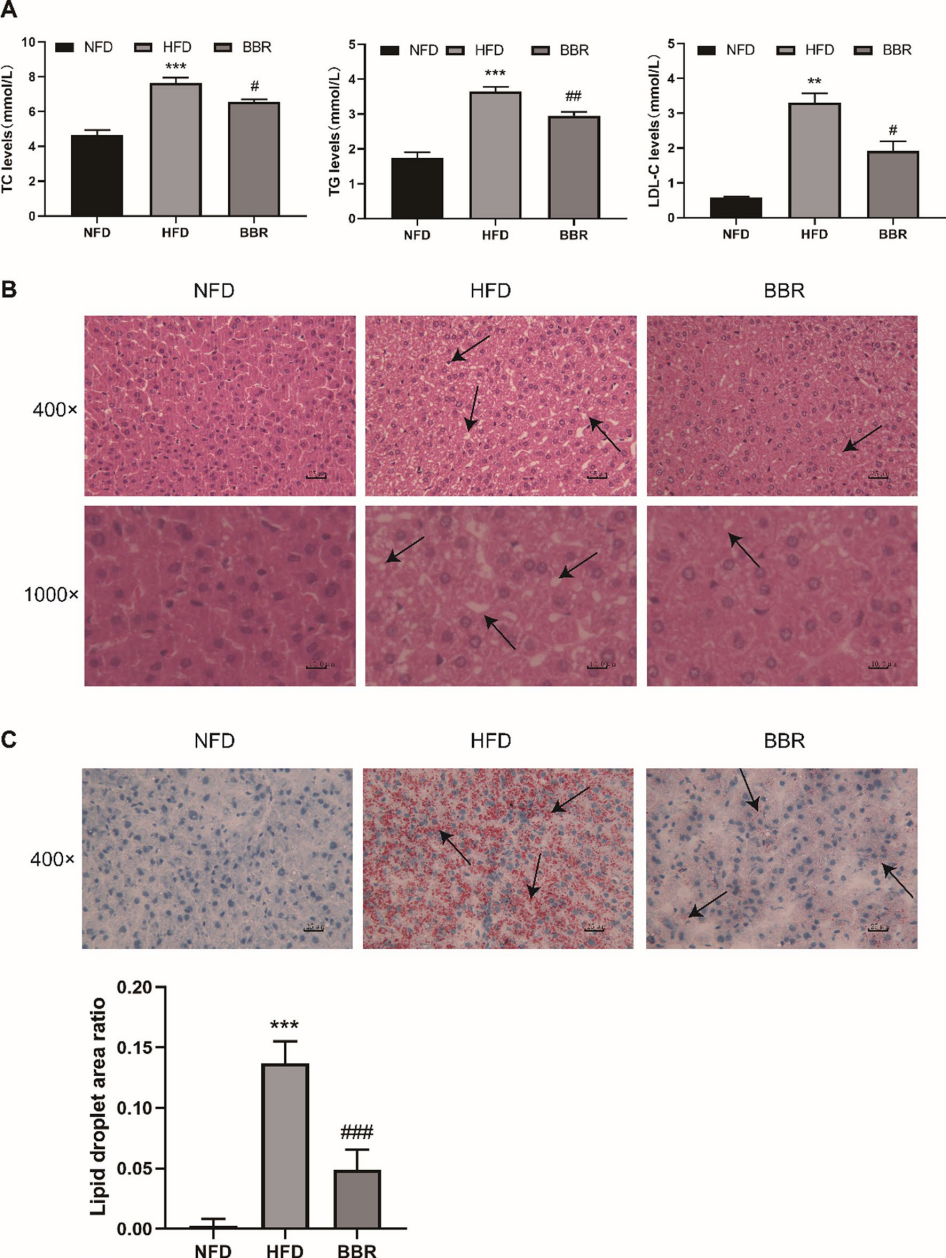
Effects of BBR on serum TC, TG and LDL-C concentrations (**A**). Compared with NFD group, ^*^p < 0.05, ^**^p < 0.01, ^***^p < 0.001; Compared with HFD group, ^#^*p* < 0.05, ^##^*p* < 0.01). Data are presented as mean ± SD (n = 6). Liver H&E staining (magnification, 400 × and 1000 ×) (**B**). Liver Oil red O staining (magnification, 400 ×) and the quantification of lipid droplet area ratio assessed by Oil red O staining (**C**). Black arrows indicate the cytoplasmic vacuolation and the aggregation of red lipid droplets, respectively. Compared with NFD group, ^***^p < 0.001; Compared with HFD group, ^###^*p* < 0.001)

The main characteristic of HLP was abnormal levels of serum TC, TG, LDL-C. As shown in Fig. 2A. The hamsters of HFD group had sharply increased serum TC, TG and LDL-C levels compared with NFD group. However, there was a significant reduction of serum TC, TG and LDL-C levels compared to those in the HFD group after BBR administration. The changes in body weight by HFD and BBR treatment were shown in Fig. S1 (Additional file 1).

### Hepatic histological analysis

The HE staining results of the hamster liver in each group are shown in Fig. 2B. The hamster hepatic cells in the NFD group have regular shapes, clear cell boundaries, and no fat droplets in the cytoplasm. Hepatocytes in the HFD group were swollen and inconsistent in size, and a large number of fat vacuoles were seen in the cytoplasm of different sizes, while fat vacuoles were reduced in the BBR group. For Oil Red O staining, the area ratio of lipid droplet in each visual field was determined by Image-Pro Plus 6.0 with red-dyed as positive staining for statistical analysis. As shown in Fig. 2C, the hamsters fed a HFD showed more extensive red-dyed lipid droplets with a higher percentage of lipid droplets. Interestingly, the proportion of lipid droplets was significantly decreased in the BBR group. These histological results implied that BBR could reduce lipid droplet accumulation in liver tissue, which alleviated the degree of hepatic steatosis.

### Lipidomics analysis

#### Lipidomics data analysis

**Fig. 3:**
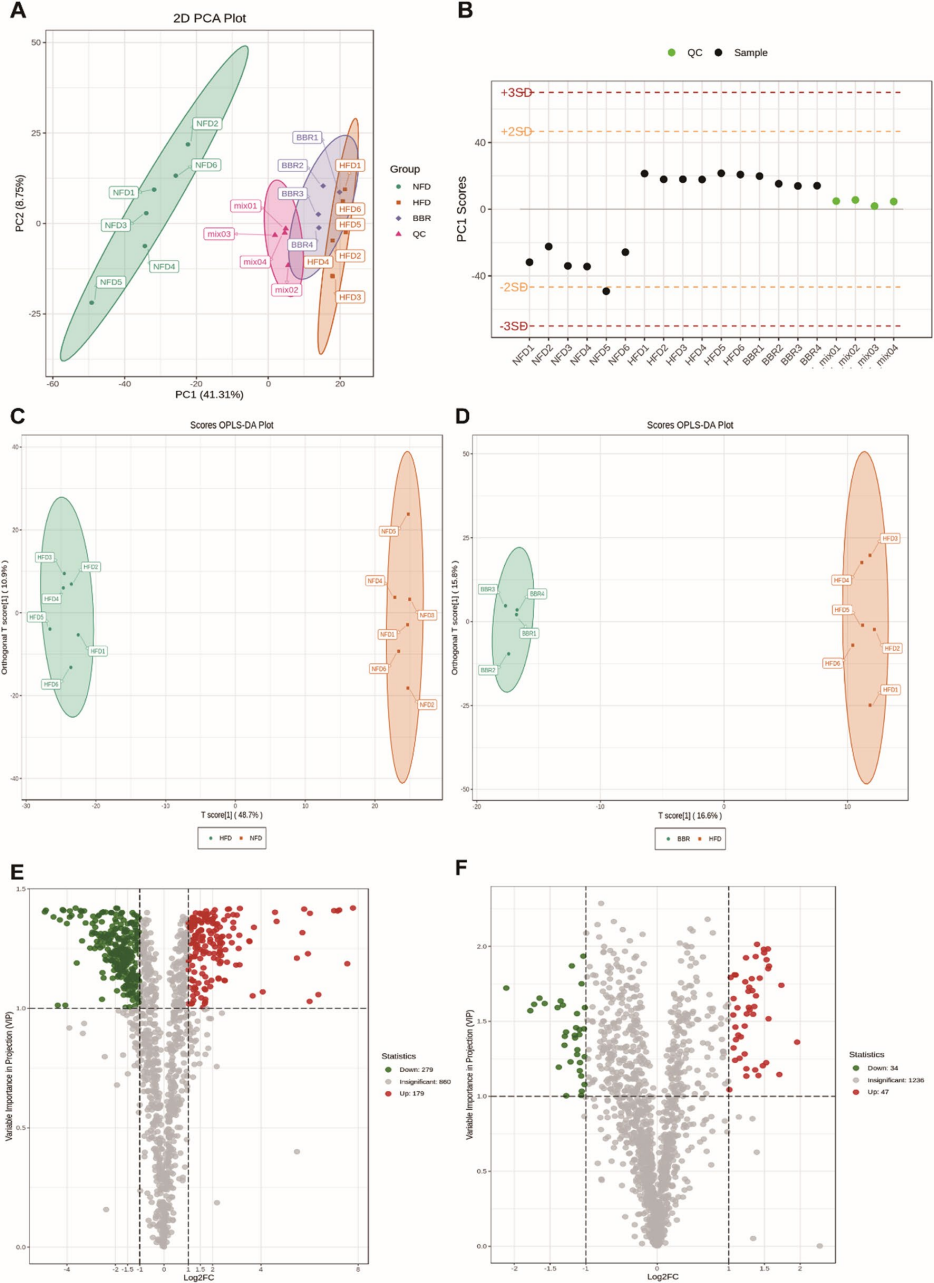
Lipidomics analysis of liver samples. Principal component analysis (PCA) score plots of hepatic lipid profiling among all groups (**A**). Score plots of PCA analysis of QC samples along PC1 axis (**B**). OPLS-DA scores plots **C**, **D** and volcano plot **E**, **F** analysis of NFD vs HFD and HFD vs BBR, respectively

The unsupervised PCA model was used to observe the dispersion trend. Quality control (QC) samples are used to verify the repeatability and stability of the instrument. PCA scores (Fig. 3A) showed that QC samples had obvious clustering, indicating that the instrument was functioning well. PCA plots also showed an evident separation trend between NFD group and HFD group, and the BBR group was closer to NFD group. The results indicated that there was a significant difference between NFD group and HFD group in the level of lipid metabolites, and the level of lipid metabolites tended to the normal level after BBR administration, suggesting that BBR has the effect of reducing the abnormal lipid metabolites induced by HFD. Based on the ion peaks detected in each sample, the QC samples were monitored by the PCA model established above to determine whether the instrument state was stable. As shown in Fig. 3B, the PC1 of the quality control samples was within the normal range of plus or minus 3 standard deviations (SD). The OPLS-DA analysis was applied to determine the lipid profile changes in the HLP model. The validation parameters of two multivariate OPLS-DA models included fitness (R2X = 0.596 and R2Y = 0.998) and predictability (Q2 = 0.982) of NFD vs HFD (Fig. 3C), and fitness (R2X = 0.982) 0.416 and R2Y = 0.999) and predictability (Q2 = 0.548) of HFD vs BBR, which illustrated that the model had the good fitness and could be regarded as a predictable model (Fig. 3D). Volcano plot analysis was used to screen out the lipid biomarkers candidates based on the criteria of FC ≥ 2 or ≤ 0.5 and VIP ≥ 1, which showed that 458 significantly different lipid species were identified in NFD vs HFD (Fig. 3E) and 81 lipids were significantly changed in HFD vs BBR (Fig. 3F).

#### Identification of the biomarkers

**Fig. 4:**
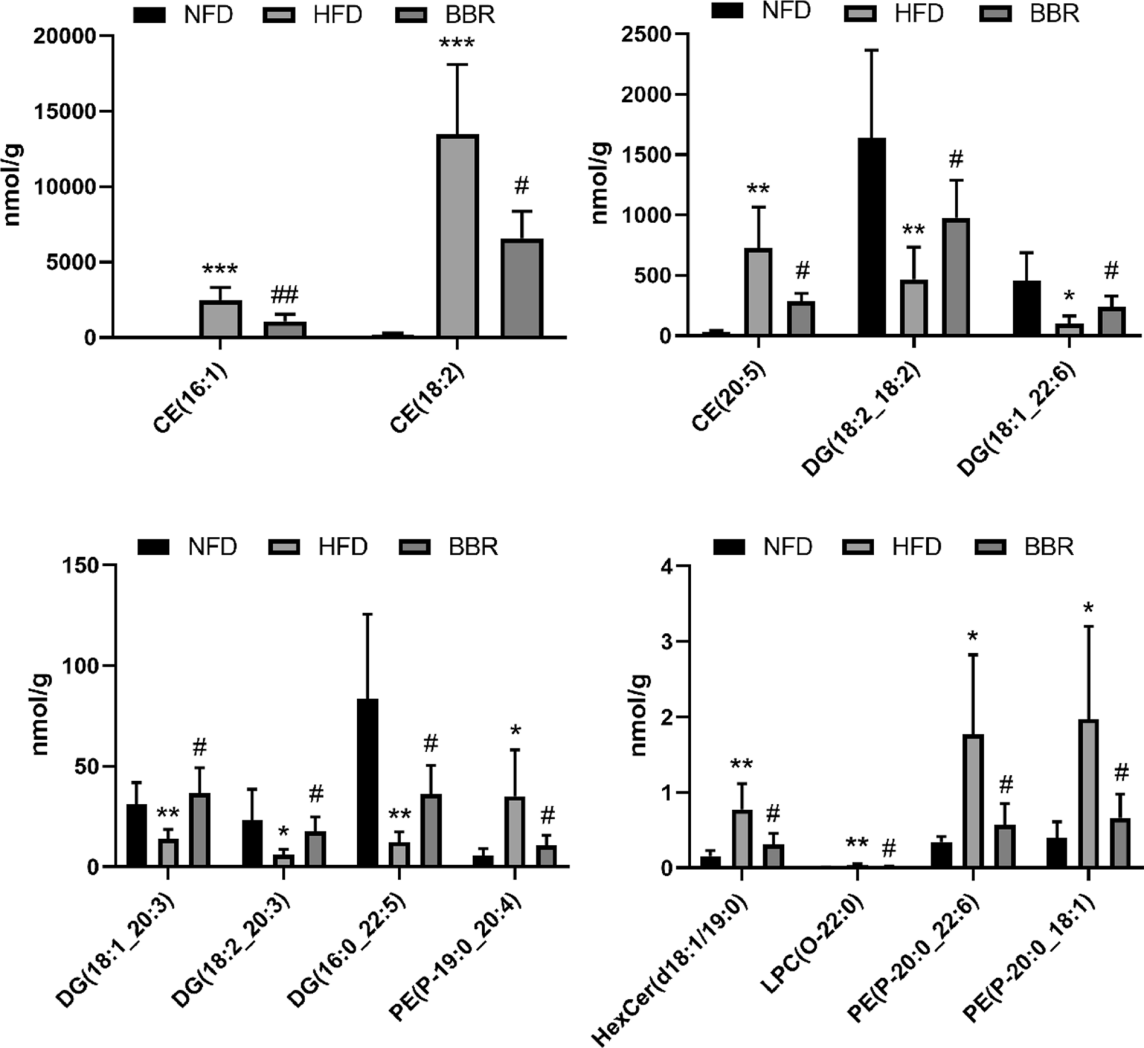
The potential lipid biomarkers responsible for anti-HLP effects of BBR. A total of 13 significantly differential lipid species were selected out by using the criteria of FC ≥ 2 or ≤ 0.5, VIP ≥ 1 and *p* < 0.05. *CE* cholesteryl esters, *PE* phosphatidylethanolamine, *LPC* lysophosphatidylcholine, *HexCer* Hexosylceramide. Compared with NFD group, ^*^p < 0.05, ^**^p < 0.01, ^***^p < 0.001; Compared with HFD group, ^#^*p* < 0.05, ^##^*p* < 0.01). Data are presented as mean ± SD (n = 6)

As shown in Fig. 4, we screened out the final potential lipid biomarkers among the three groups (p < 0.05). The contents of three CEs (CE(16:1), CE(18:2), CE(20:5)), HexCer (HexCer(d18:1/19:0)), LPC-O (LPC(O-22:0)) and three PE-Ps (PE(P-20:0_22:6), PE(P-20:0_18:1), PE(P-19:0_20:4)) in the HFD group were significantly enhanced compared to the NFD group, while BBR supplementation significantly decreased the content of these lipids (p < 0.05). While the five DGs (DG(18:2_18:2), DG(18:1_20:3), DG(18:2_20:3), DG(16:0_22:5), DG(18:1_22:6)) in the HFD group was significantly lower than that in the NFD group, while the content of them in the BBR group was significantly increased. Therefore, these results suggest that the 13 lipids, including 3 CE, 1 HexCer, 1 LPC-O, 3 PE-P, and 5 DG, might be the potential biomarkers for the lipid-lowering effect of BBR.

#### Pathway analysis

**Fig. 5:**
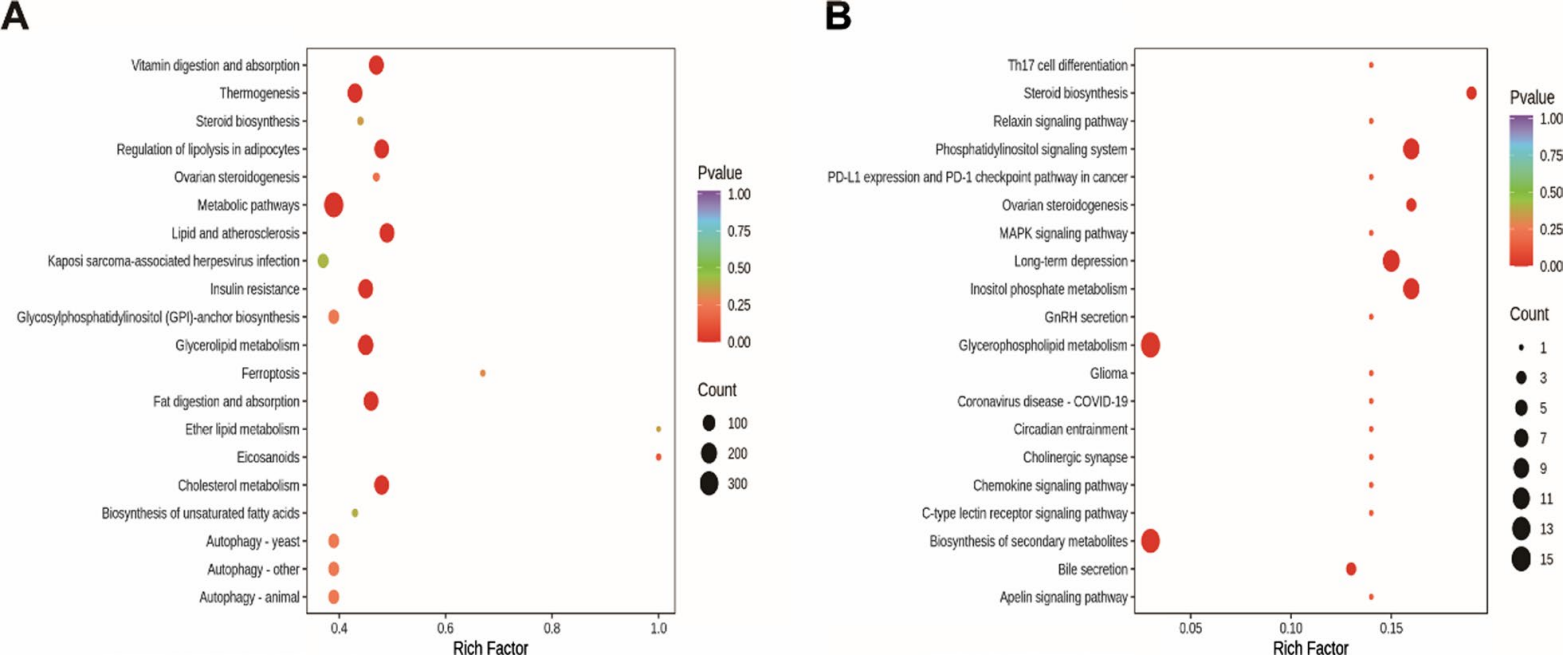
Lipid metabolic pathway analysis based on significantly differential lipid species in NFD versus HFD **A** and HFD versus BBR (**B**). Degree of enrichment was analyzed by a rich factor, P-value and the number of lipid metabolites that enriched in each pathway. The size of bubble means the amount of significantly differential lipid species which are enriched in this pathway, and the point with different gradation of color represents the scope of P-value. The higher value of rich factor stands for the higher degree of enrichment, and the lower P-value represents the more significant degree of enrichment

Pathway enrichment analysis can provide some indication of biochemical and signal transduction pathways in which differential lipids may be involved. As shown in Fig. 5A, B, the hamsters in HFD group were clearly damaged the pathways of thermogenesis, regulation of lipolysis in adipocytes, fat digestion and absorption, steroid biosynthesis and cholesterol metabolism compared with the NFD group, whereas BBR supplementation ameliorated the pathways of steroid biosynthesis, phosphatidylinositol signaling system, inositol phosphate metabolism and glycerophospholipid metabolism.

### Network pharmacology

#### Targets prediction of BBR against HLP and lipid metabolite targets

**Fig. 6:**
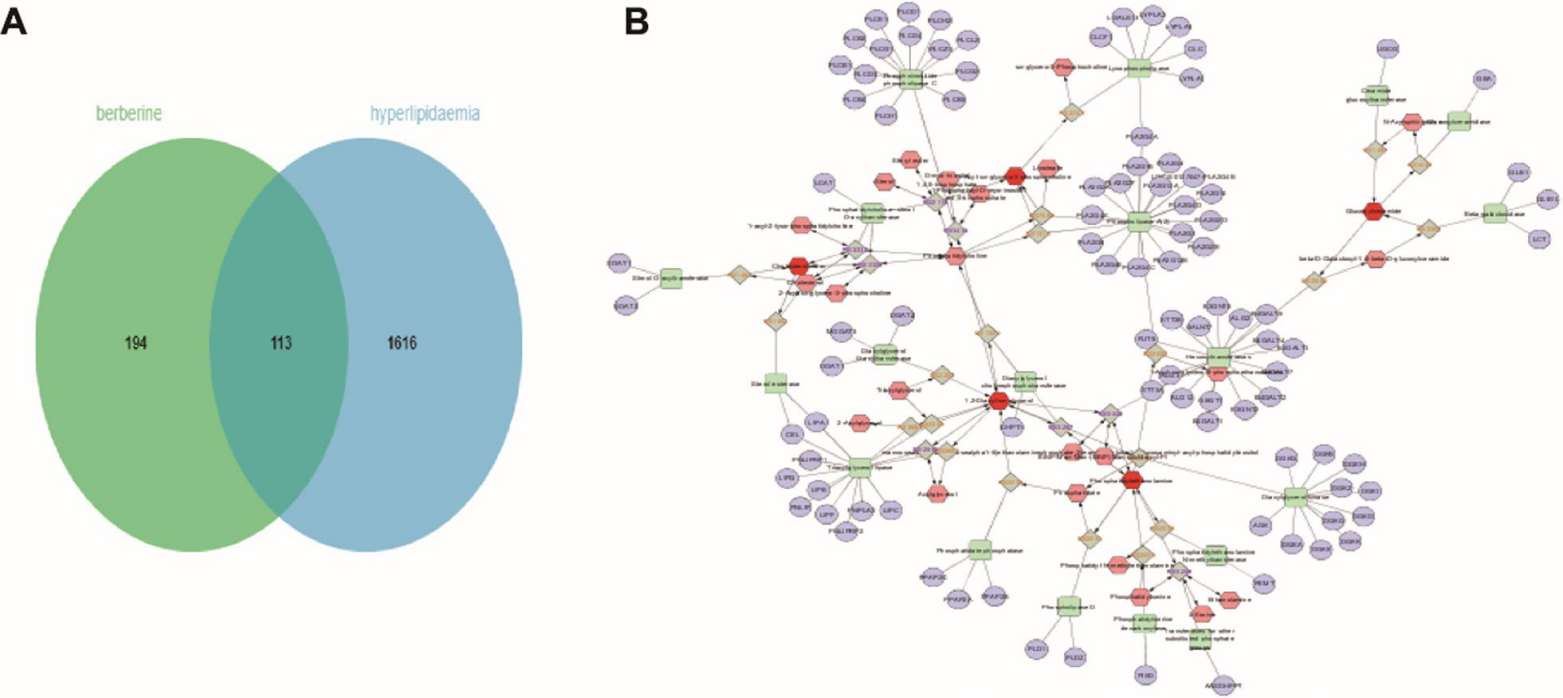
Screening of targets of BBR against HLP and lipid metabolite targets. Distribution of BBR and HLP target genes (**A**). BBR-regulated lipid metabolite-reaction-enzyme-gene network (**B**)

In the present work, a total of 307 targets of BBR were predicted based on the TCMSP database, Swiss Target Prediction, Pubchem Server and PharmMapper Server. The target genes related to HLP were searched in the GeneCards Database, DrugBank, OMIM Database and DisGeNET database, which were taken the intersection with the 1776 active targets. After the deletion and integration of false positive targets and repeat targets, 113 potential targets of BBR against HLP were obtained (Fig. 6A). In addition, the BBR-regulated lipid metabolites were introduced into Metscape to generate a metabolite-reaction-enzyme-gene network (Fig. 6B). Finally, 94 lipid biomarker targets were obtained (Table 2).

**Table 2:** 94 lipid biomarker target information

ALG3
PEMT
LYPLA1
CEL
B3GNT2
FUT9
B4GALT7
PLCD3
LYPLA2
CLC
PLA2G4E
DGKK
DGKA
DGKB
DGKG
DGKH
DGKQ
B3GNT6
STT3B
PLCH1
PLCL2
PLCB1
CLCF1
LYPLA3
PISD
PLA2G4F
PLA2G2D
GBA
GBGT1
B4GALT1
GLB1
PLA2G4D
LGALS13
PLA2G2E
MOGAT3
STT3A
LCAT
LCT
LIPA
LIPB
LIPC
PLA2G3
PLCE1
GALNT7
PLA2G1B
PLA2G2A
PLA2G4A
PLA2G5
PLCB2
PLCB3
PLCB4
PLCD1
PLCG1
PLCG2
PLD1
PLD2
PNLIP
PNLIPRP1
PNLIPRP2
AGK
CHPT1
AASDHPPT
PLA2G2F
SOAT1
UGCG
ALG12
GLB1L
PNPLA3
PLA2G12A
PLA2G6
PLA2G10
SOAT2
PLA2G12B
DGAT2
PLCD4
LIPF
DGKZ
DGKE
DGKD
ALG2
PLA2G4C
PPAP2A
PPAP2C
PPAP2B
LOC100137047-PLA2G4B
DGAT1
B4GALT4
B4GALT3
B4GALT2
PLCZ1
DGKI
B4GALT5
LIPG
PLCH2

#### Network analysis

**Fig. 7:**
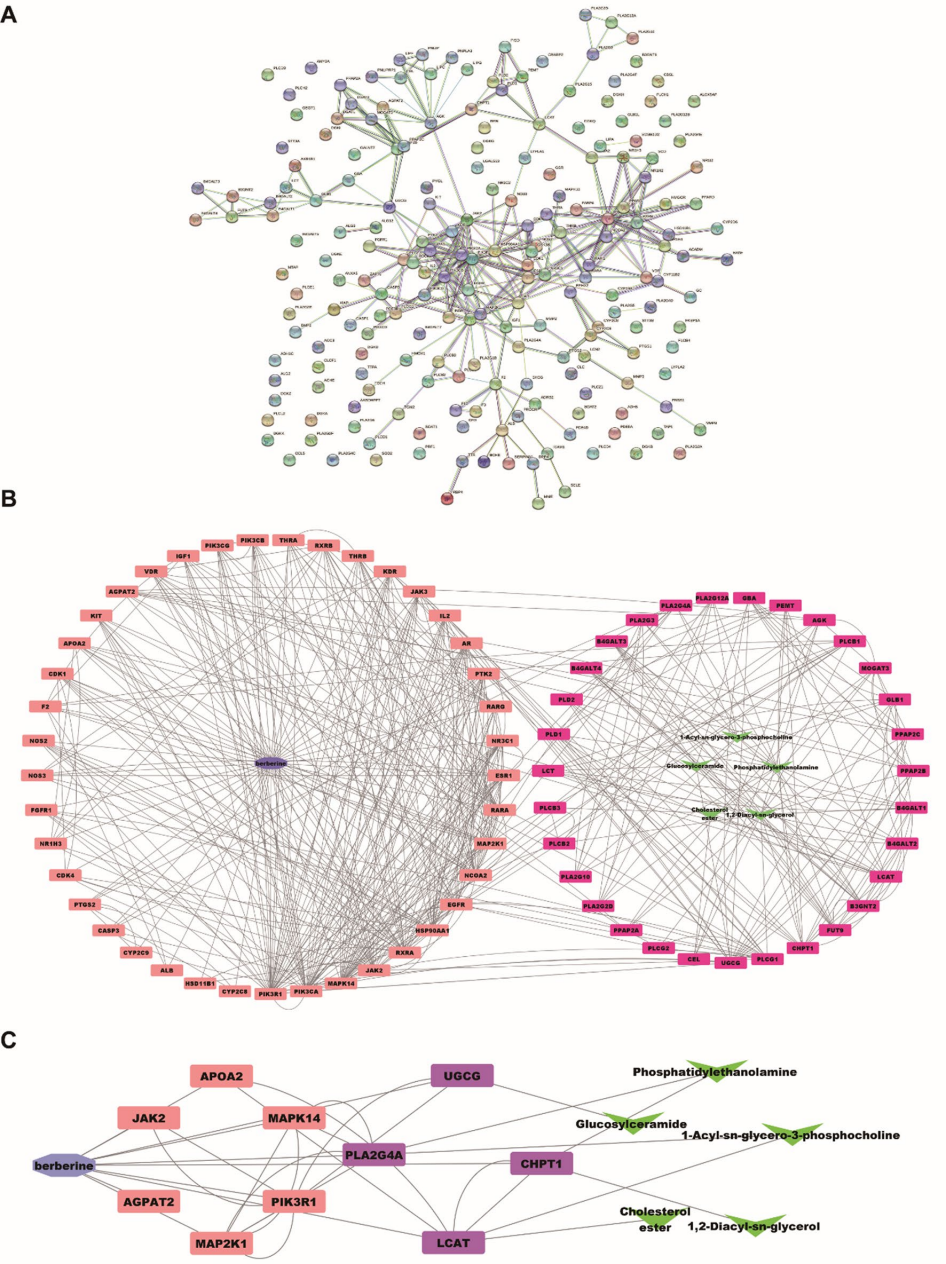
PPI network of BBR and lipid markers targets against HLP (**A**). The “BBR-target-metabolite” network (**B**). The “screened key BBR-predicted target-lipid target” network. (Blue: BBR, Orange: predicted-targets, Purple: lipid metabolite targets, Green: metabolites) (**C**)

We linked the 94 lipid metabolite targets with 113 predicted targets of BBR against HLP using STRING 11.5 to build the PPI network (Fig. 7A). According to the results of the PPI network analysis, the connected targets was established to clarify the underlying mechanism of BBR treating HLP. The BBR-metabolite-target network was generated by Cytoscape 3.9.1. The two important parameters of betweenness and degree were used to evaluate the key lipid metabolites and the key targets regulated by BBR. As shown in Fig. 7B, we screened 37 targets belonging to BBR against HLP and 44 targets belonging to lipid metabolite targets based on the criterion that both degree and betweenness value were greater than the median. Based on the above screening, the targets with the top 10 of betweenness values were selected as the key targets (Fig. 7C). The main predicted targets of BBR were JAK2, AGPAT2, MAP2K1, PIK3R1, MAPK14 and APOA2, and the predicted targets linked lipids targets were UGCG, PLA2G4A, LCAT and CHPT1.

### Further validation of the targets

**Fig. 8:**
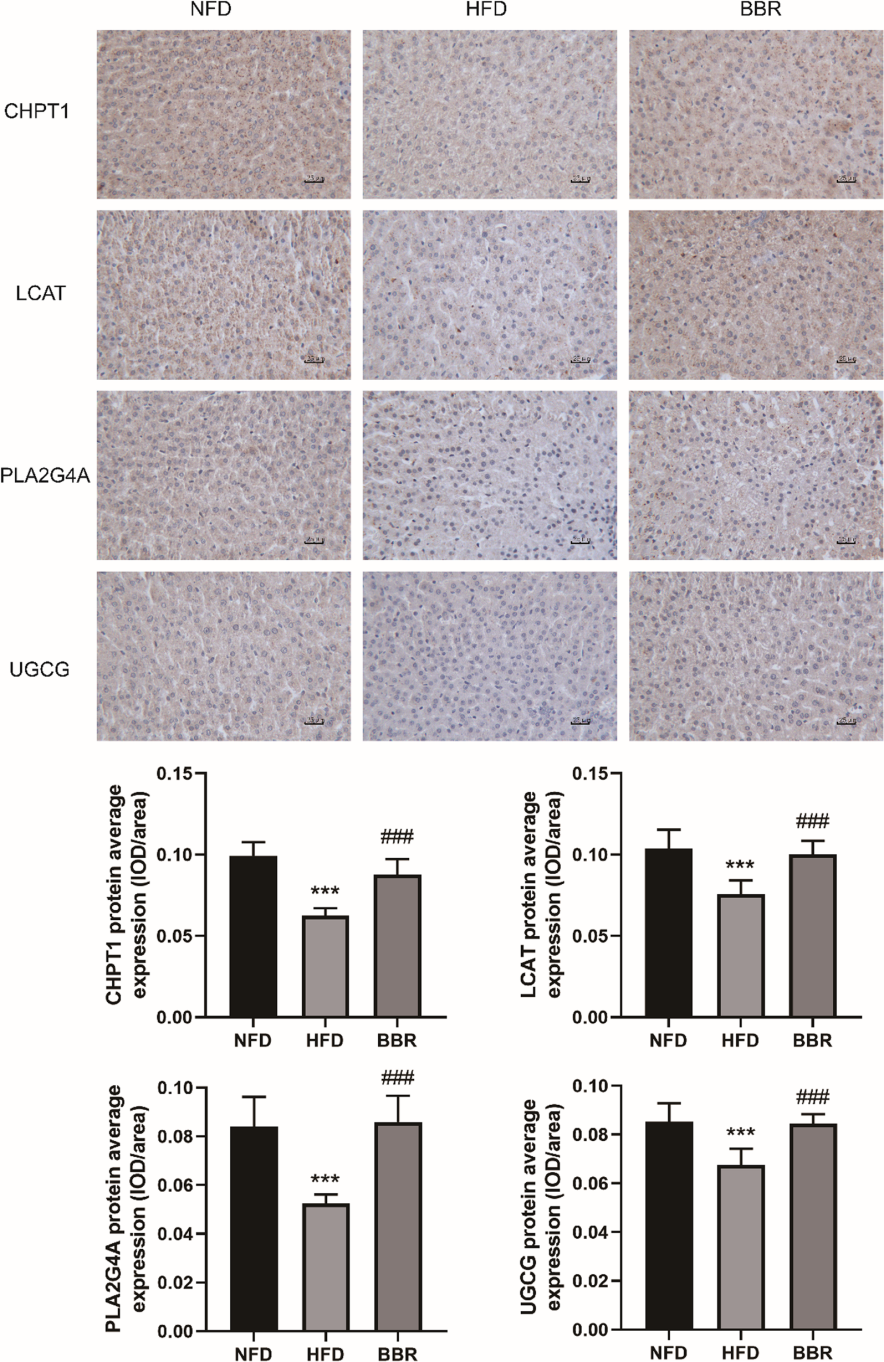
The effect of BBR on CHPT1, PLA2G4A, LCAT and UGCG protein of liver tissues in hyperlipidemic hamsters (Immunohistochemistry, 400 ×). Compared with NFD group, ^***^p < 0.001; Compared with HFD group, ^###^*p* < 0.001). Data are presented as mean ± SD (n = 6)

The further validation of the targets in liver tissues of different groups were identified by immunohistochemical staining. The mean optical density in each visual field was determined by Image-Pro Plus 6.0 with brown-yellow as positive staining for statistical analysis. As shown in Fig. 8, the mean optical density of CHPT1, LCAT, PLA2G4A and UGCG expression in HFD group was significantly lower than that in NFD group and the BBR group.

## Discussion

Berberine has cholesterol lowering effects and aids in the prevention of metabolic diseases [17–19]. Since the anti-hyperlipidemia of BBR has been well studied in earlier work, which suggested that LDLR was identified as a target of BBR [4]. And the similar effect was observed in our study, we found that BBR significantly increased the levels of TC and TG in the serum of LDLR-deficient hyperlipidemic hamsters (Additional file 1: Fig. S2), while BBR could significantly reduce the levels of TC and TG in the wild-type hyperlipidemic hamsters (Fig. 2A). In this study, we developed a novel integrated strategy combining lipidomics with network pharmacology to explore new molecular targets of BBR in the treatment of HLP.

**Fig. 9:**
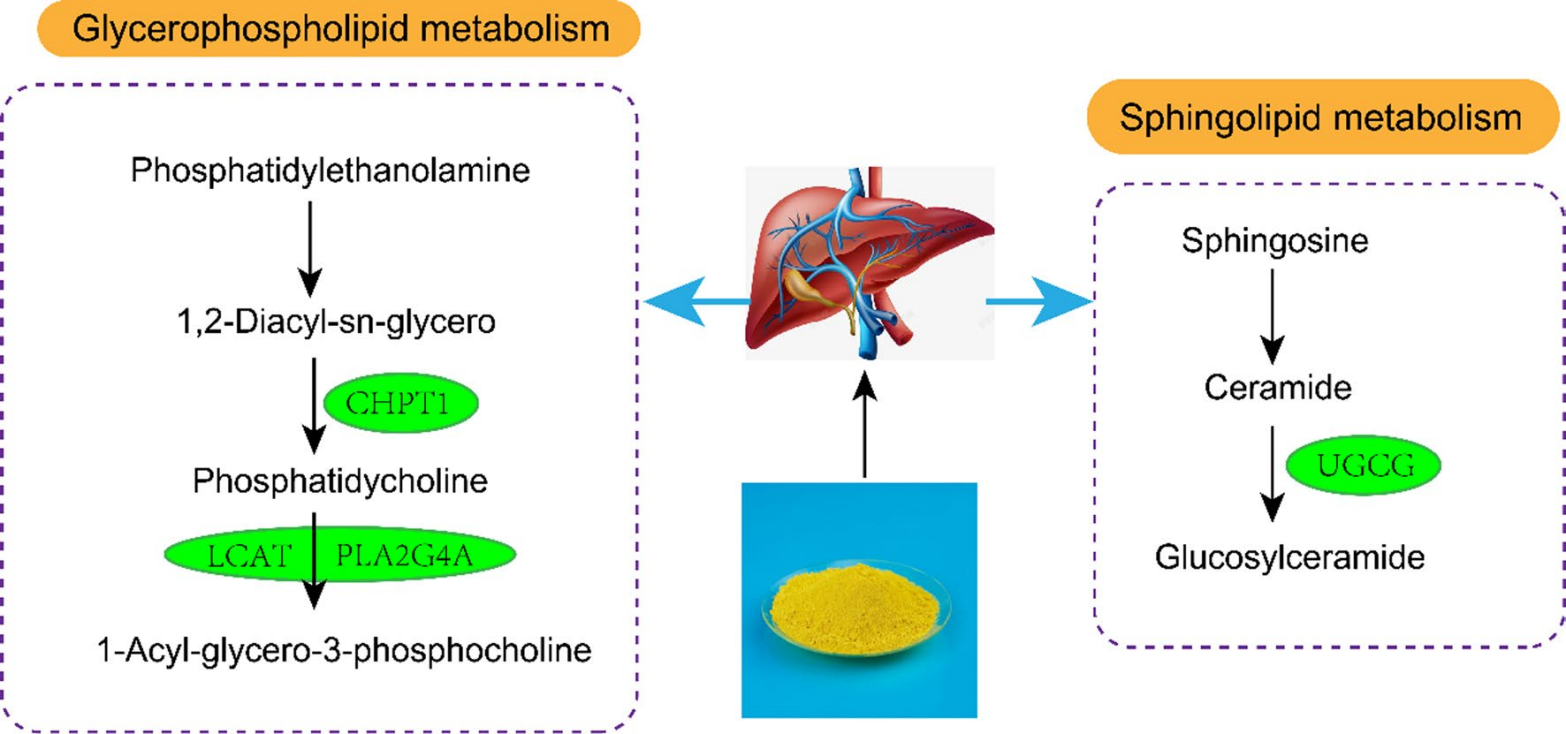
Hypothetical molecular mechanisms of BBR treating HLP

Lipidomics and network pharmacology results showed that the effect of BBR against HLP was involved with regulating glycerophospholipid, sphingolipid, steroid metabolism and 10 targets (JAK2, AGPAT2, MAP2K1, PIK3R1, MAPK14, APOA2, UGCG, PLA2G4A, LCAT, CHPT1). In summary, BBR improved HLP in model hamsters by targeting multiple targets through multiple metabolic pathways. The specific action mechanism of BBR on HLP treatment was shown in Fig. 9. We adopted immunohistochemical analysis to further verify that the BBR anti-HLP molecular targets of CHPT1, PLA2G4A, LCAT and UGCG, which have not been reported.

Glycerophospholipids, including phosphatidylcholines (PCs) and phosphatidylethanolamines (PEs), are ubiquitous and important biological components. Choline phosphotransferase 1 (CHPT1), an enzyme responsible for encoding the final enzyme of the PCs synthetic pathway, can catalyze PCs synthesis and regulates choline metabolism [20]. CHPT1 acts as a choline phosphotransferase in the final step of phosphatidylcholine synthesis, catalyzing the transfer of phosphocholine from CDP-choline to diacylglycerol, simultaneously releasing CMP and phosphatidylcholine. Lysophosphatidylcholine (LPC) is hydrolyzed by phospholipase A2 (PLA2) and a molecule of fatty acid is released from glycerophospholipids. As it contains a hydrophobic hydrocarbon chain and a polar phosphate group, which can destroy the cell membrane and rupture red blood cells, resulting in hemolysis. A study showed that PCs could contribute to proliferative growth and programmed cell death [21]. LPC was reported to be a pro-inflammatory lipid mediator that stimulated human monocytes to produce IL-1β and involved in the pro-inflammatory process of chronic diseases [22]. In this experiment, the concentration of LPC (O-22:0) in liver of hamsters in HFD group was higher than that in NFD group, suggesting that abnormal lipid metabolism was involved in the unbalance of anti-inflammatory system, which may be an important mechanism for the occurrence and development of HLP. Levels of LPC (O-22:0) was decreased after BBR treatment, which indicated that overactive LPC (O-22:0) might be inhibited by BBR.

LCAT (cholesterol ester acyltransferase) is the only key enzyme that catalyzes the esterification of plasma cholesterol. It is involved in the reverse transport process of cholesterol from plasma to liver, and its deficiency leads to abnormal lipid deposition, increased oxidative stress and atherosclerosis [23, 24]. In this study, it was observed that the levels of three kinds of CEs decreased after BBR treatment compared with HFD group, suggesting that BBR may play a lipid-lowering role through LCAT.

Udp-gluco ceramide glucosyltransferase (UGCG) is the first rate-limiting step in the glucoceramide biosynthetic pathway, promoting glucoceramide production and thereby increasing iNKT cell activity and cytokine production to reduce insulin resistance. Overproduction and/or accumulation of ceramide and ceramide metabolites, including glucosylceramides, can lead to insulin resistance [25]. The results of this experiment showed that HexCer(D18:1/19:0) content in BBR group was lower than that in HFD group. Therefore, BBR may reduce insulin resistance in HLP by balancing UGCG enzyme expression.

## Conclusion

This study develops a novel integrated strategy combining lipidomics with network pharmacology to explore the therapeutic mechanism of BBR in the HLP model. Lipidomics results showed that 13 different lipids were regulated by BBR, including cholesterol esters, diglycerides, glycosphingolipids, lysophosphatidylcholine, and phosphatidylethanolamine. Pathway analysis suggested that the protective effect of BBR may be related to the regulation of steroid biosynthesis, phospatidylinositol signaling system, inositol phosphate metabolism and glycerophospholipid metabolism. The results of lipidomic and network pharmacology analysis showed that CHPT1, PLA2G4A, LCAT and UGCG were expected as the lipid-linked targets of BBR against HLP, which were further verified by immunohistochemical analysis. Taken altogether, this study preliminarily elucidated the pharmacological mechanism of BBR on HLP through lipidomics and network pharmacology, which contributes to the further efficiency evaluation of BBR.

## Supplementary Information

**Additional file 1: Fig. S1.** The changes in body weight by HFD and BBR treatment. Compared with NFD group, *p<0.05, **p<0.01, ***p<0.001; Compared with HFD group, #p<0.05, ##p<0.01). Data are presented as mean ± SD (n=6).** Fig. S2.** The levels of TC and TG in the serum of LDLR-deficient hyperlipidemic hamsters by HFD and BBR treatment. Compared with L-NFD group, *p<0.05, **p<0.01; Compared with L-HFD group, #p<0.05).

## Data Availability

All the data generated and analyzed during this study are included in the manuscript.

## Abbreviations

BBR: Berberine (BBR)
HLP: Hyperlipidemia
TC: Total cholesterol
TG: Triglyceride
LDL-C: Low-density lipoprotein-cholesterol
CE: Cholesteryl esters
PE: Phosphatidylethanolamine
LPC: Lysophosphatidylcholine
HexCer: Hexosylceramide
