# Supplementary material for: Integrated lipidomics and network pharmacology analysis to reveal the mechanisms of berberine in the treatment of hyperlipidemia
Source: J Transl Med. 2022 Sep 8;20:412. doi: 10.1186/s12967-022-03623-0 (PMC9461205; doi:10.1186/s12967-022-03623-0)
Supplement: Supplementary file 1 — Additional file 1: Fig. S1. The changes in body weight by HFD and BBR treatment. Compared with NFD group, *p<0.05, **p<0.01, ***p<0.001; Compared with HFD group, #p<0.05, ##p<0.01). Data are presented as mean ± SD (n=6). Fig. S2. The levels of TC and TG in the serum of LDLR-deficient hyperlipidemic hamsters by HFD and BBR treatment. Compared with L-NFD group, *p<0.05, **p<0.01; Compared with L-HFD group, #p<0.05). [file 12967_2022_3623_MOESM1_ESM.docx]

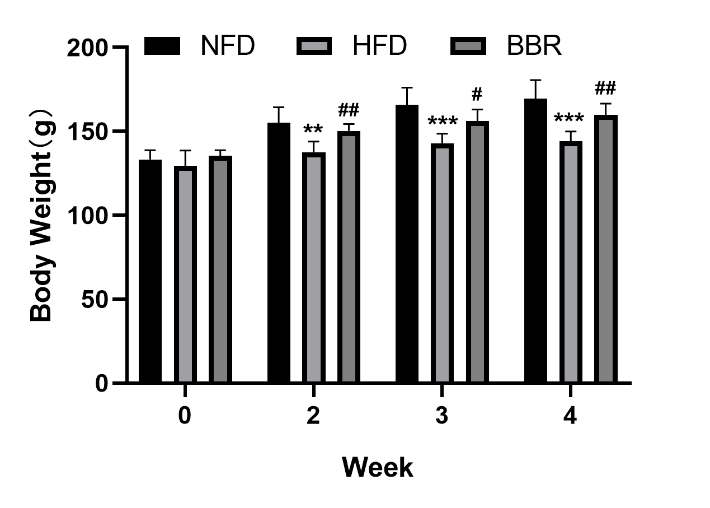


**Fig. S1** the changes in body weight by HFD and BBR treatment. Compared with NFD group, ^*^*p*<0.05, ^**^*p*<0.01, ^***^*p*<0.001; Compared with HFD group, ^#^*p*<0.05, ^##^*p*<0.01). Data are presented as mean ± SD (n=6).


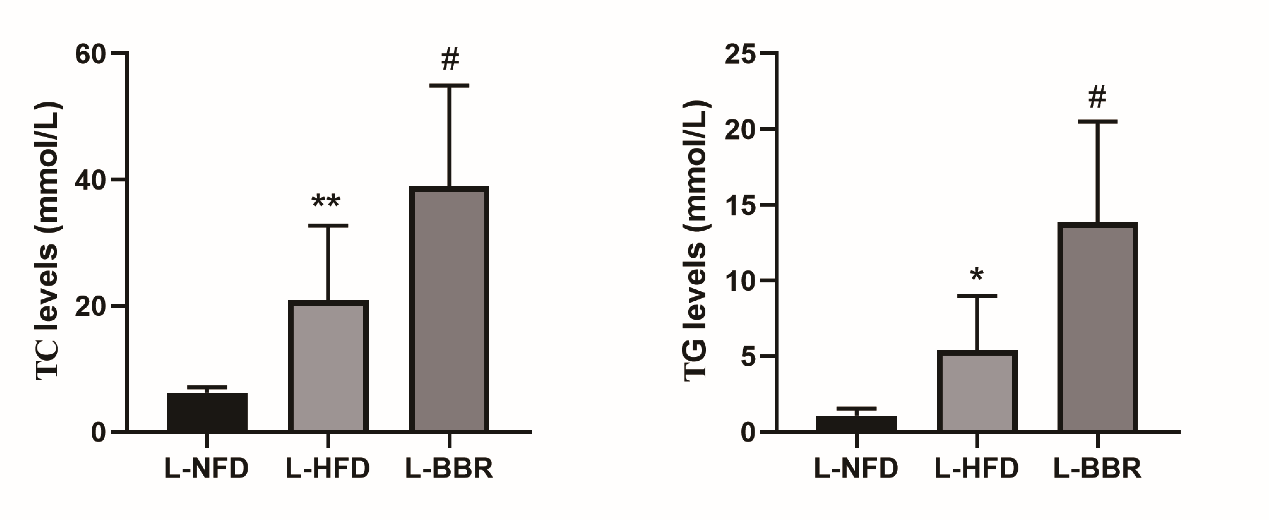


**Fig. S2** the levels of TC and TG in the serum of LDLR-deficient hyperlipidemic hamsters by HFD and BBR treatment. Compared with L-NFD group, ^*^*p*<0.05, ^**^*p*<0.01; Compared with L-HFD group, ^#^*p*<0.05).
